# A Guide to *Ex Vivo* Biodistribution Studies with Radiotracers in Rodent Models

**DOI:** 10.1007/s11307-025-02055-8

**Published:** 2025-10-28

**Authors:** Surachet Imlimthan, Cesare Berton, Sophie Poty, Jason P. Holland, Mirkka Sarparanta

**Affiliations:** 1 Department of Chemistry, University of Helsinki, 00014 Helsinki, Finland; 2 Department of Chemistry, University of Zürich, 8057 Zurich, Switzerland; 3 Institut de Chimie Moléculaire de L’Université de Bourgogne (ICMUB), UMR CNRS 6302, Université de Bourgogne, 21000 Dijon, France

**Keywords:** *Ex vivo* biodistribution, Radiolabeled tracers, Radiopharmaceuticals, Biodistribution protocol, Biodistribution calculator

## Abstract

Preclinical biodistribution studies are required at an early stage of radiopharmaceutical development to determine tracer pharmacokinetics in clinically relevant animal models of human diseases. When combined with quantitative analysis from non-invasive imaging, biodistribution experiments provide essential data on the uptake, retention, binding specificity, metabolism, and clearance of radiotracers in both target and non-target tissues. Most research groups have developed in-house protocols to perform these studies in a reproducible manner. However, there is a general lack of consistency in how different groups carry out biodistribution experiments. In addition to practical differences that occur during tissue collection (for example, washing and blotting dry the tissue, perfusion, sampling site for a given tissue, etc.), other aspects of biodistribution experiments, which often vary include the methods used for calibrating the injected activity, the processes used to calculate mass normalized tissue uptake (i.e. percentage of injected dose per gram [%ID g^−1^] or percentage of injected activity per gram of tissue [%IA g^−1^]) values, differences in data processing and statistical analyses (particularly error propagations and calculation of tissue contrast ratios), and variations in how the methods and data are reported and interpreted. This variability hinders the direct comparison of datasets produced at different laboratories. Here, we present a comprehensive guideline for conducting *ex vivo* biodistribution experiments with radiotracers in rodent models. An open source, freely accessible online biodistribution calculator and associated spreadsheet are provided which can be employed to compute the percent of injected dose per gram of tissue (%ID g^−1^), standardized uptake value (SUV by mass), and target-to-background tissue contrast ratios. Finally, advice concerning biodistribution data presentation and statistical analysis are given to help the reader harness the full power of *ex vivo* biodistribution studies in radiotracer development.

**Supplementary Information:**

The online version contains supplementary material available at 10.1007/s11307-025-02055-8.

## Introduction

**Fig. 1:**
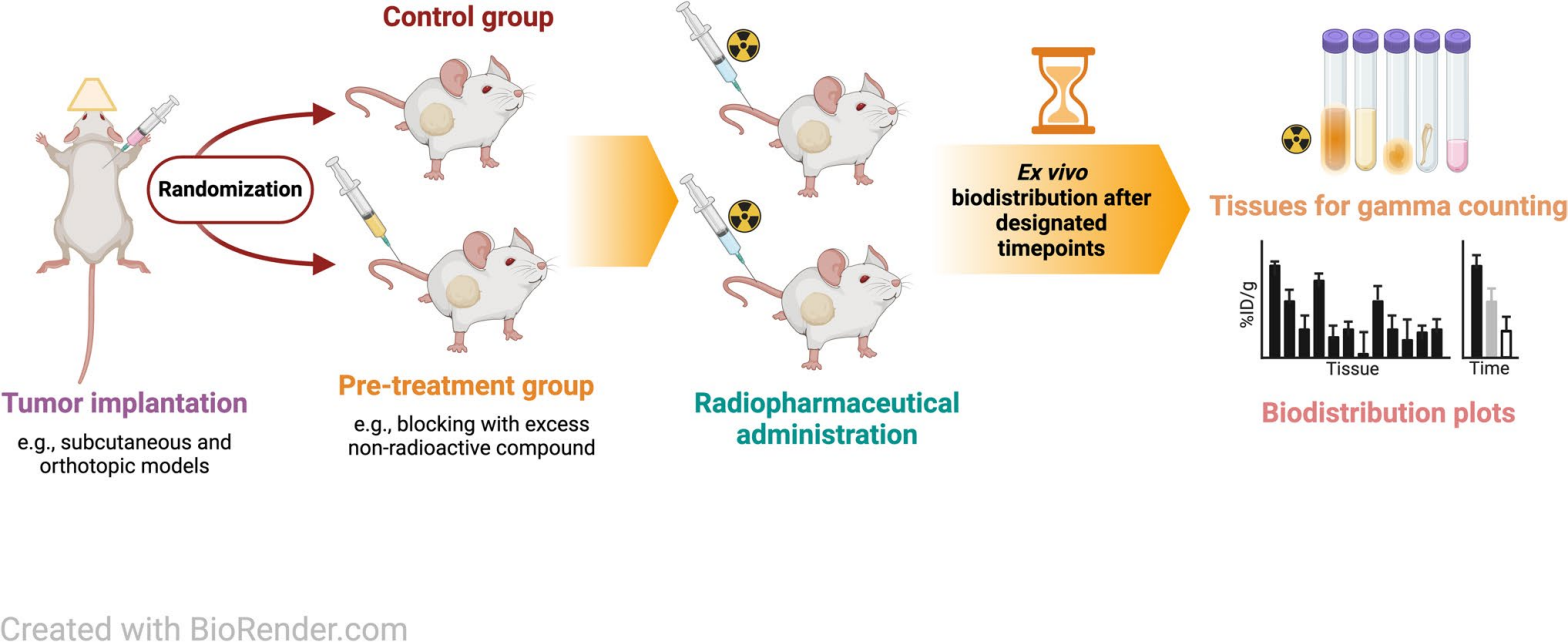
Schematic representation of a general framework for *ex vivo* biodistribution studies in a tumor model. First, the tumors are established by subcutaneous or orthotopic implantation of tumor cells. Second, when the tumor is appropriately developed (typically > 100–200 mm^3^ in volume, as normally measured by external Vernier calipers), animals are randomized into study groups, such as those receiving different treatments or the baseline or blocking groups. Third, the radiopharmaceutical is administered to the animals typically by intravenous (i.v.) tail vein injection. Finally, animals are euthanized at predetermined time points, and selected tissues are collected. Tissue samples are weighed and the accumulated activity in each sample is counted (using e.g., a gamma counter, scintillation counter, or dose calibrator). Data are then processed to determine the amount of activity, relative to the injected amount, per unit mass of tissue, which is tabulated and plotted for interpretation, or further proceeded to calculate contrast ratios between target lesions and background tissue. Created in BioRender. Imlimthan, S. (2025) https://BioRender.com/7xg8m49

Understanding the biodistribution and pharmacokinetics of radiotracers in preclinical models is a key step toward the successful development and clinical translation of radiopharmaceuticals for applications in diagnostic imaging and molecularly targeted radiotherapy [1, 2]. In addition to longitudinal non-invasive imaging by using positron emission tomography (PET) or single-photon emission computed tomography (SPECT) methods, *ex vivo* biodistribution studies provide high fidelity in the quantification of radiotracer accumulation across target and background tissues, and the necessary input for preclinical dosimetry [3]. Biodistribution studies are deceptively simple, with the core component of the experiment involving sacrificing groups of animals at defined time points after radiotracer administration, collection of tissue samples, and the measurement of activity (Fig. 1) [4, 5]. Yet, practical differences in how the experiments are designed and performed, and in data processing, can have a dramatic impact on the outcome, interpretation, and reproducibility of the results. Standardized nomenclature and data practices are essential for advancing radiopharmaceutical development and ensuring study consistency. Following existing guidelines [6, 7], our work supports broader standardization efforts. As artificial intelligence (AI) driven analysis relies on well-structured datasets, adherence to established guidelines and methods enhances interoperability and reproducibility, a key to support AI’s expanding role in data interpretation and discovery [8].

Biodistribution assay results are typically expressed as the percentage of the injected dose of activity per unit mass of tissue (%ID g^−1^), though some prefer the equivalent percentage of injected activity %IA g^−1^ to avoid confusion with dosimetry. In this manuscript, we use %ID g^−1^, the standard unit for the past decades, but a shift to %IA g^−1^ is expected. The data can then be used to determine target-to-background contrast ratios and the standardized uptake values (SUV). The value of SUVs has been questioned, and different numerical calculations based on body weight, area, or body-mass indices can be used [9]. In our work, SUVs calculated by body weight represent a reasonable and experimentally facile way of comparing tissue associated activity across a series of mice within a group, and between groups. However, a critical problem is how to determine the administered activity accurately using the instruments available in a typical radiolabeling or imaging suite. Dose calibrators – the tool of choice in clinical practice – are extremely sensitive to sample geometry and not the most accurate way of determining activity doses at the sub-MBq range typically used in preclinical studies [10]. Consistent sample orientation and geometry help mitigate this issue, but small activity doses used in preclinical studies may still affect accuracy. Regular calibration and fixed measurement geometry are essential for reliable dose calibrator readings. On the other hand, the gamma counters are far more sensitive than dose calibrators but can become saturated at the activity levels used, making measurement of the administered dose without dilution impossible. Dilution introduces additional reproducible and irreproducible errors which decrease the accuracy of the measurement. As an alternative, we and others, have turned to the most sensitive and accurate instruments in the radiolabeling lab, the analytical balance (with accuracy down to < 0.01 mg or lower for microbalances) and the gamma counter, to determine the injected activity. By measuring the injected mass of formulated radiotracer solution given to each individual animal and applying a correction for residual activity that is left in the syringe, an accurate amount for the activity administered in each mouse can be determined based on a weighed standard prepared from the injected activity. However, the dose calibrator (if accurately calibrated for the geometry used) is not obsolete in this setup, as it can be used to easily corroborate the correct level of activity in the drawn doses to reveal, for example, any sticking of the radiotracer onto the syringe surface.

**Fig. 2:**
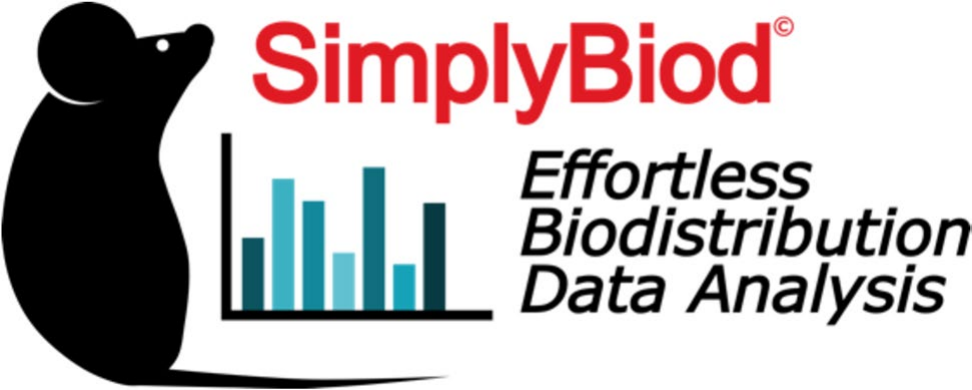
The *SimplyBiod*^*©*^ web-based biodistribution calculator. The tool is open-source and freely accessible at https://simplybiod.streamlit.app/. It features automatic decay correction for all activity values to a common temporal reference, supports over 80 radionuclides, and includes a comprehensive graphing section for quantitative analysis and visualization

This guide provides a step-by-step protocol to *ex vivo* biodistribution studies, covering data processing, interpretation, and special considerations like studies with alpha emitters, transcardial perfusion, and collection of challenging tissue samples. Our aim is to standardize experimental procedures, data collection, analysis, and reporting. Ultimately, the implementation of standardized procedures is compliant with the 3Rs principle, leading to a reduction in animals used and facilitating the reuse of datasets with confidence. The accompanying online biodistribution analysis tool (*SimplyBiod*^*©*^, Fig. 2) is designed to offer a free, open-source, and user-friendly interface that helps streamline data analysis for *ex vivo* biodistribution studies. Furthermore, we foresee that the online tool can be expanded to an online *ex vivo* biodistribution database, where relevant data can be deposited in accordance with the FAIR (Findable, Accessible, Interoperable, Reusable) principles and compliant with the essential Open Access requirements of most funding agencies today.

## *Ex Vivo* Biodistribution Protocol for Radiopharmaceuticals

This protocol describes a step-by-step procedure for carrying out *ex vivo* biodistribution, with key considerations (for example, critical points, extra care, and recommendations) that must be considered when conducting the research. Important to note is that this general protocol is independent of the animal model for which we anticipate that the reader is best placed to make the appropriate selection for their research. The methods for carrying out an *ex vivo* biodistribution study presented here cover the aspects of dose calibration, tissue harvesting, radioactivity counting, and data processing.

### General Requirements and Considerations

#### Safety and Biohazards

All experiments involving living organisms and radioactivity must follow institutional radiation protection and biosafety guidelines. Personal protective equipment (PPE), including lab coats, gloves, safety glasses, face masks, and personal dosimeters, is required. All procedures must be conducted in biosafety level-appropriate laboratories, with proper waste disposal, including radioactive, biological, and sharp waste management.

#### Ethical Considerations

The study must comply with local ethical authorities, e.g. the Institutional Animal Care and Use Committee (IACUC). All procedures should minimize pain and distress, with anesthesia and analgesia used as needed. The study protocol must be reviewed and approved by the respective local authority to ensure humane and justified animal use.

#### Study Design

Giving comprehensive guidance on the study design within the scope of this guide is not feasible, as researchers best understand their biological targets and models. However, variability in design and parameters across studies remain a major challenge for result comparability. Minimizing the variation by careful consideration of not only the radiotracer parameters (e.g. molar activity, radiochemical purity, formulation) but the study design itself would be the way forward and can likely supersede the variation in tissue collection and radioactivity measurement as a main source of differences in results obtained at different sides. Aspects of experimental design to consider and report in studies include the methods used for the determination of an appropriate animal number (e.g. power analysis), the strain, age, sex and breeder of the animals used, pathogen status, the housing conditions (e.g. lighting rhythm, humidity, temperature, bedding, feed, water, enrichments), the timing of the experiment relative to the time of day and the estrous cycle of the animals, the anesthesia and other drugs used, post-operative pain management and its efficacy, body temperature, dosing in awake versus anesthetized animals, and euthanasia and tissue collection methods. Additionally, if cell lines are used to generate tumors, they should be authenticated and tested free of common pathogens like *Mycoplasma* periodically. A good illustration of the importance of the study design on the biodistribution of a radiotracer can be found in the work of Fueger and co-workers, showing the drastic impacts of animal handling to the biodistribution and tumor-to-organ ratios obtained with 2-[^18^F]fluoro-2-deoxyglucose ([^18^F]FDG) [11]. Furthermore, the ARRIVE (Animal Research: Reporting of *In Vivo* Experiments) guidelines [12, 13] provide an evidence-based framework for designing and reporting reproducible animal experiments. The requirement to follow the ARRIVE checklists is becoming prevalent in journals and considering them during planning greatly facilitates reporting.

### Materials and Equipment

- Syringe preparation sheet (Supplementary Information, Table S1).
- Injection sheet (Supplementary Information, Table S2).
- Dose calibrator. The researcher should ensure that the dose calibrator is regularly calibrated by using an appropriate activity standard to obtain an accurate calibration factor for the ionization chamber for the specific radionuclide and geometry used in the experiment [14]. Additionally, linearity checks should be conducted to verify that the measurement range adequately encompasses the activity levels used in the experiment.
- Calibrated analytical balance (accurate to at least four decimals preferred). When there are numerous samples to analyze, investing in a high-throughput analytical balance with semi-automated operation (e.g., with gesture sensors or keyboard shortcuts for closing and opening the balance door, and recording of weight) linked to an Excel spreadsheet is highly recommended. It should be noted that radiation safety should be prioritized to maintain ALARA (As Low As Reasonably Achievable) principles, particularly during dose measurement in syringes. If feasible, a dedicated balance should be placed behind appropriate shielding. Additionally, the distance between the hand and the radioactive dose can be maximized by using long-handled forceps to handle the syringes.
- Pre-weighed tubes with caps for the tissue collection that are compatible with the sample holders of the gamma counter used.
- Calibrated automatic gamma counter for radioactivity quantification with presets for the appropriate energy window of the used radioisotope, decay correction to the start of the measurement.
- Microsoft Excel-based biodistribution calculator (Supplementary Spreadsheets)

### General Procedures

#### Radiotracer Administration and Organ Collection

After radiosynthesis, purification, and characterization of the radiotracer to confirm standard quality control parameters, including the radiochemical and radionuclidic purity, the chemical identity of the product, the molar activity of the sample (*A*_m_ in application relevant units e.g. GBq μmol^−1^), the radiotracer is formulated for injection. First, an aliquot of the purified radiotracer containing the required dose in nanomoles and/or activity (in MBq) is added to a suitable volume of a biocompatible formulation buffer (typically sterile isotonic saline or phosphate-buffered saline, PBS, but other formulations can be used depending on the chemical properties of the radiotracer). ♦**Critical**♦ Sufficient volume of formulated radiotracer should be prepared to draw at least *n* + 1 syringes where *n* is the number of animals to be injected. The additional dose is required for preparation of a counting standard, which is later used to calibrate the amount of injected material (see below). For example, if a study containing *n* = 5 mice requires administration of 100 μL of a radiotracer per animal, an aliquot of the purified radiotracer containing sufficient nmol or activity to prepare *n* + 1 doses should be added to the formulation buffer and the total volume diluted to (6 × 100 μL = 600 μL). After preparing the formulated radiotracer, syringes (typically insulin syringes 27–30 gauge needles, 0.5 mL total volume) are drawn, each containing 100–250 μL desired injected volume. ♦**Caution**♦ Many radiotracers at nmol levels exhibit pronounced non-specific binding to plastic syringes, thereby affecting the accuracy of the dose administered. This is frequently the case for lipophilic small-molecule and some peptide-based radiotracers. To reduce non-specific binding, pretreatment of the syringe barrel to reduce the sticking of the radiotracer might be needed. This may be accomplished by, for example, pre-incubating the syringes with 1% (w/v) bovine serum albumin (BSA), which has been found to be beneficial for many radiotracers. The BSA solution can be prepared in sterile isotonic saline or PBS. The syringes should be filled with the BSA solution and incubated for at least 2 h in the refrigerator. If needed, the syringe needles can be thoroughly rinsed with a small amount of formulated buffer or saline to remove any excess BSA before use. This helps ensure that only the bound BSA remains while preventing any interference with the radiotracer and reducing the risk of contaminating the dose. ♦**Critical**♦ The measurement of activity, time, and weight of a syringe both before and after injections must be done using the same dose calibrator, clock, and balance, respectively. After drawing the syringe, any excess liquid on the exterior of the injection needle and outside of the syringe barrel should be wiped away with a sterile tissue paper to avoid contamination.

##### Before Injection

Each syringe must be marked with an identifying number on the plunger/barrel and cap. After drawing the syringes, the radioactivity, time of measurement, and full weight of the loaded syringe must be measured and recorded on the syringe preparation form (see Table S1 for an example sheet).

##### Radiotracer Administration

The animal identification, tail/ear marking, the injection time, and other potential identifiers/experimental parameters should be recorded on the injection sheet (see Table S2 for an example sheet).

##### After Injection

The residual activity in each syringe should be measured and recorded with the time of measurement and empty weight of the syringe (see Table S1). Recording the residual activity and mass of a syringe after administration must be conducted within a timeframe that aligns with the decay properties of the radionuclide under study. Failure to record the remaining activity in an appropriate time window may lead to unaccounted variability in the determination of the administered dose.

♦**Extra Care**♦ Note, our protocol and the data processing template assume that the injections are done without problems, meaning 100% of the administered dose was injected as a single bolus directly into the blood stream. If the injection was missed, or some amount of activity was lost during the procedure, the researcher should make a note in the comments section Table S1. Where necessary, manual corrections for fractions of activity that were not injected can be made by measuring the residual activity in the syringe and adding to this the measured activity of contaminated materials that were used during the injection procedure for the individual animal. Other routes of administration can also be used without changes to the basic principles of data collection and processing. Corrections can also be made later for activity that was injected into the tail. ♦**Recommendation**♦ If significant adsorption of radiotracers is suspected, mass-based calculations of the injected dose may not be accurate. In such cases, residual activity can be measured by using a dose calibrator or gamma counter to ensure accurate quantification of the injected dose.

At designated time points after radiotracer administration, animals are euthanized using the methods approved in the project license (e.g., CO_2_ asphyxiation, exsanguination under anesthesia, etc.), and the tissues of interest are collected (see Table S3 for an example list of tissues). ♦**Critical**♦ In general, a blood sample is first taken through cardiac puncture under anesthesia without opening the abdominal cavity, and the tumor is then collected to avoid contamination of tissues with the blood in the subsequent harvesting steps. In the head-and-neck region, skin/ear flap, brain, and occipital bone are collected. After laparotomy, internal organs (from top to bottom) are harvested in the following order: gallbladder (with caution; see tips and tricks section below), heart, lung, liver, spleen, pancreas, stomach, small intestine, large intestine, adrenal gland, kidneys, fat, other internal tissues of interest, urine (directly from the bladder or CO_2_ chamber), muscle and tibia from the hind limb, and tail (injection site). Internal organs should be rinsed briefly with lukewarm water or buffered saline to remove potential radioactive contamination from blood and then blotted dry on a damp paper towel to remove any excess liquid on the surface that could interfere with the actual organ weight. The content inside organs of the GI tract, such as stomach and intestines may be separated for counting if it is suspected to interfere with the result interpretation. ♦**Extra Care**♦ It should be noted that the blot-dry procedure cannot be used with delicate organs and biofluids, such as urine, blood, brain, and gallbladder, which are best collected and placed directly into the counting tubes. Moreover, excess fat and any adjacent tissues adhering to collected organs must be completely removed before placing the tissue sample into the allocated pre-weighed biodistribution tube for accurate weight.

Following the collection procedure, tissues are transferred to a pre-weighed and labeled collection tubes, and the tubes are sealed with the assigned cap (also measured together with the tube during pre-weighing). The weight of the tissue can be calculated by subtracting the weights of the tube filled with the organ and the pre-weighed empty tube. Note that the tubes and caps have significant variation in weight. Weighing a few tubes to obtain an average weight for the collection tubes is not accurate and can lead to large errors in the final data analysis that will compromise the validity of the experiment. ♦**Recommendation**♦ In order to avoid mixing the caps, both the tube and the cap should be labeled with the same assigned code with alcohol-resistant marker, or barcode stickers can be used depending on the number of samples. It is recommended to have a list of organs with the coding system close at hand to double-check before transferring an organ to the corresponding tube. Adhering to the same order of tissue collection across different studies reduces the likelihood of errors. Furthermore, the weight of an empty tube and that of a tissue-filled tube must be measured on the same calibrated balance. ♦**Extra Care**♦ To avoid cross-contamination of activity from previous procedures, blood-contaminated gloves should be changed before transferring the organ to the tube. It is recommended to use forceps to transfer the samples and to clean them between samples. Also, the dissection station and tools should be decontaminated after each animal. If the tissue-filled tubes are not weighed straightaway after gamma counting and must be stored in a freezer for any reason, ensure that they are equilibrated to room temperature and that there is no water condensed on the tube exterior before weighing.

#### Counting Standards Used for Accurate Quantification

1. One standard syringe of radiotracer formulation is drawn from the same sample and at the same time as the syringes used for radiotracer administration in animals. The weight, activity, and time both before and after injection of the standard syringe are recorded in syringe preparation form (Table S1). The standard syringe is handled in precisely the same way as the syringes that are injected into the animals. The only difference is that the standard syringe is injected into a sample of water to create a diluted solution from which counting standards are prepared.

For preparation of the stock solution used to create counting standards, an empty 15-mL conical centrifuge tube (with cap) is weighed, and the weight is recorded. Approximately 10 mL of water is added to the tube, and the weight (tube + water) is recorded. The injection formulation (for example 100–250 μL) in the standard syringe is then injected into the tube, the time recorded, and the total weight of the tube (tube + water + standard) is measured. Residual activity, time, and weight of the standard syringe after emptying the contents to the stock solution, are recorded. ♦**Critical**♦ The standard syringe should be injected into the water in a single bolus. Do not rinse the syringe by pumping the plunger up and down, as this will not be representative of the administration to animals. Moreover, to minimize non-specific binding, the 15-mL conical tubes should be treated with 1% BSA solution, following the syringe treatment protocol.

The standard dilution tube is thoroughly mixed with a vortex mixer. Approximately 1 mL of the stock solution (see step 2 above) is aliquoted into at least 4 separate, pre-weighed (including tube and cap) gamma counting tubes to create four counting standards. The weight of the counting standards (tube + cap + approximately 1 mL standard dilution) is recorded. Note, these counting standards are later used to obtain (after appropriate correction for decay and dilution factors) a calibration factor in terms of the total counts per minute per gram of formulation (CPM g^−1^ of the injected activity), which is used to calculate the total injected activity per animal based on the injected mass. ♦**Optional**♦ As an alternative, researchers could at this step also create a serial dilution to simultaneously check the linearity of the gamma counter and obtain the same calibration factor. Gamma counter calibration factors can drift daily, causing non-linearity and underestimating activity in high-retention organs like the kidneys and liver. To ensure accuracy, verifying linearity across expected activity levels using known standards helps to identify and correct any deviations for reliable biodistribution data.

2. The standard and tissue tubes are now ready to be measured with the gamma counter. It is recommended that researchers count tubes in a fixed sequence to avoid errors and facilitate the input of the counting data to the biodistribution calculator. For example, the samples could be arranged as follows: background (in quadruplicate), counting standards (in quadruplicate), and tissue tubes in the sequence of tissue collection (e.g., blood, tumor, gallbladder, heart, lungs, liver, spleen, pancreas, stomach, kidneys, small intestine, large intestine, fat, muscle, bone, brain, bladder, etc.). The activity in the sample is typically expressed in counts per minute (CPM) with values decay corrected to the start of the measurement. ♦**Recommendation**♦ It is recommended that the gamma counter is set to perform automatic decay correction where all corrected CPM values are decay-corrected to the start of the gamma counting measurement. In principle, any time point can be selected but since the gamma counter automatically corrects the counted samples to one time point, it is practically easier to then forward corrected the injected doses to the same time point, rather than back correcting all values to, for example, the time of administration. If the injected activity is relatively high, it is best to recount the samples at a later point to ensure that they are not saturating the gamma counter detector. The appropriate time between the biodistribution sample collection and gamma measurement needs to be determined by the user to ensure that all counted samples lie within the linear range of the gamma counter.

#### Data Processing and Biodistribution Calculator

Along with this protocol, we provide two tools for analyzing *ex vivo* biodistribution data with a comprehensive radionuclide list, streamlining data processing and improving consistency across labs. One, a downloadable Microsoft Excel-based biodistribution calculator (Supplementary Spreadsheets: blank template and template with filled examples) allows users to input protocol data. Table S4 (Supplementary Information) outlines its logical input flow with at least 35 entries. Input parameters are grouped and assigned cell numbers with relevant notes. In the biodistribution template, light orange cells require experimental data, while light blue cells auto-populate via linked formulae. Calculations for %ID g^−1^ and SUV are given in the Supplementary Information. Once data are entered, %ID g^−1^ and SUV values are calculated for individual animal and group averages. Graphical plots display in columns P to AG, with target-to-background ratios in AI to AZ. All activity values are decay-corrected to the start of the gamma counter measurement. In principle, any time point can be used for decay correction, but selecting the gamma counter’s start time allows direct data entry into the spreadsheet.

Second, the *SimplyBiod*^*©*^ online tool developed in this collaboration simplifies *ex vivo* biodistribution analysis, reducing errors and streamlining data processing. Free, open-source, and user-friendly interface, it supports over 80 radionuclides, automatic decay correction, and comprehensive graphing. Built in Python with Streamlit dashboard, it’s accessible on any browser-enabled device. The tool is available at https://simplybiod.streamlit.app/, with source code on GitHub repository (github.com/cecebert/SimplyBiod). The site offers extensive features like a broad selection of radionuclides, automated decay correction, automatic calibration factor calculations (in CPM MBq^−1^), and intuitive tissue selection. Additionally, its plotting section enables side-by-side comparisons between multiple animals and tissues. In its current iteration, *SimplyBiod*^*©*^ processes user inputs only within the active session, ensuring security, with all data erased upon closing. In future developments, we will consider implementing a free database for data storage and cross-study comparisons.

## Special Considerations for Biodistribution Studies

### Whole-Body Measurement

**Fig. 3:**
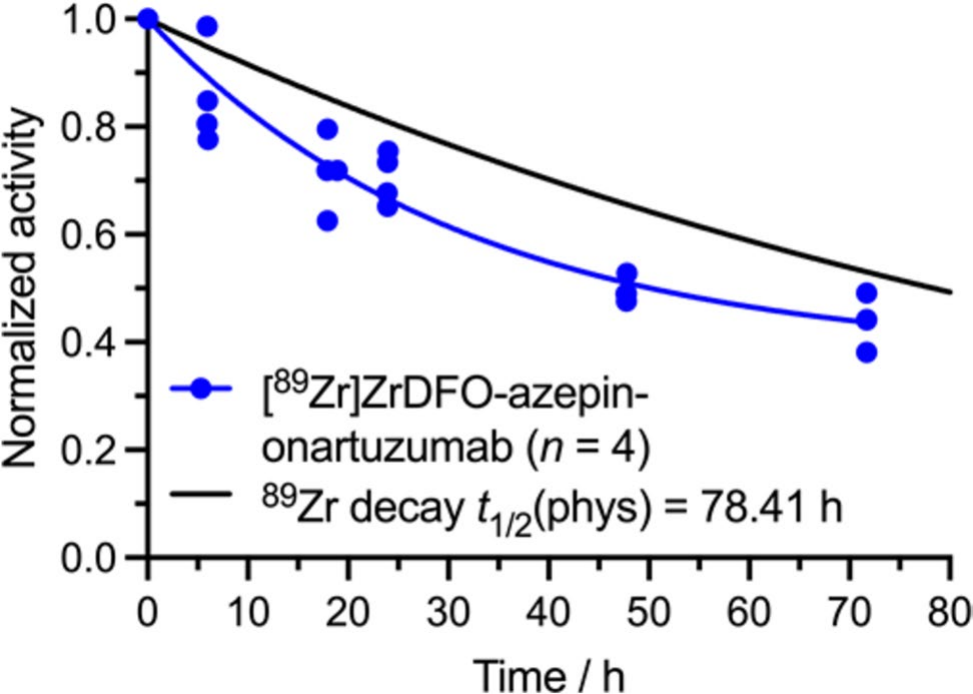
Plot showing the experimentally measured effective half-life of a ^89^Zr-radiolabeled antibody (blue data points) in athymic nude mice recorded by measuring the total activity in each individual animal *versus* time using a dose calibrator. Data are modeled with a one-phase exponential decay (blue line). The physical decay of ^89^Zr (*t*_1/2_ = 78.41 h) is shown by the black line. Radiotracers that are excreted from the body faster will show a larger difference between the measured effective half-life and the physical half-life of the radionuclide

The effective half-life (*t*_1/2_(eff)/h) of radiotracer excretion is a simple, informative parameter for large group of mice. These data are recorded by measuring the total activity in each individual animal at different time points during the experiment using a dose calibrator (inserting either anaesthetized or awake animals into the counting chamber) or whole-frame imaging with a volume of interest (VOI) encompassing the entire animal. Representative data recorded by using a dose calibrator are presented in Fig. 3. Although the metabolism and excretion pathways of most radiotracers are usually unknown, we find that datasets on the measured total activity in the animals *versus* time typically fit well with a one-phase or two-phase exponential decay model. For the two-phase model, the two derived rate constants can usually be interpreted as being associated with separate distribution and elimination phases, or faster and slower pathways of excretion. For example, albumin-binding tracers typically exhibit a distribution phase before elimination, and tracers excreted via both renal and hepatobiliary routes often have faster urinary than fecal excretion. We also note that, in the absence of other data such as metabolite analysis, fitting with the simplest model is recommended [15–17]. Measuring the effective half-life of a radiotracer is useful for comparing how different chemistry (e.g., chelate, radionuclide, linker, etc.) affects the biochemical properties of radiotracers with the same biologically active vector in the same animal model.

### Tips and Tricks on Tissue Collection and Hard-to-Collect Samples

The selection of tissues to collect during an *ex vivo* biodistribution study depends on the nature of the radiotracer, including target distribution, elimination routes, and expected sites of free radiolabel accumulation, such as the bone for radiotracers labeled with fluorine-18 and zirconium-89, and the thyroid or stomach for radioiodinated tracers. Adhering to a predetermined tissue collection order (see example in Table S3) is advisable as this type of systematic procedure decreases the variation in sample weight and the risk of misplacing samples. To attain as much data as possible, 17–25 tissue samples per animal are typically collected, but this can be reduced for subsequent experiments with the same radiotracer based on low activity organs or specific research goals. Alternatively, organs like the brain can be dissected into finer segments (different brain areas). During the dissection, it is advisable to move from the more important (e.g., the tumor) or low in activity tissues to those rich in blood and higher in activity (e.g., the heart and liver) to avoid activity contamination to the instruments. Challenging but important fluids to collect for radiometabolite analysis include urine and bile. Since legislation and guidelines on allowed methods of euthanasia vary between countries, it is not possible to give generalized protocol. However, in most cases urine can be collected after euthanasia from the bladder, or non-invasively prior to euthanasia by placing the animal on a non-absorbing surface or hydrophobic collection medium, such as LabSand®. A tip for urine collection is to place the individual awake animal into a clean glass beaker that has been precooled on ice for 5 min. The animals usually urinate rapidly on placement into the beaker. Note that this procedure must be approved in the ethical license and the animal should be continuously monitored inside the cooled beaker where it remains for a maximum of 2 min. Habituation to handling is advisable. If urination has not occurred, the animal should be removed and either processed in the usual biodistribution or returned to the cage for a subsequent attempt (if experimental time points and protocols permit).

For blood and urine, the contents of the syringe should be ejected to the counting tube immediately after sampling. The gallbladder with bile (only in mice) is best collected directly after the laparotomy. The gallbladder can be found as a transparent greenish-yellow vesicle between the two top lobes of the liver. Note that rats do not have gallbladders and cannulation of the bile duct might be needed. Care must be taken to avoid puncturing the gallbladder when cutting ventral skin and muscles. The gallbladder can be lifted out holding the bile duct with fine forceps and cutting the duct. The gallbladder should be placed directly into the counting tube as contents will spill when the grip of the forceps is released. The thyroid is particularly challenging to localize and dissect in mice and is best dissected under a stereomicroscope. Care must be taken to avoid severing the arteries in the neck when dissecting the muscles to expose the trachea to prevent obscuring the view with blood.

The removal of stomach and intestinal contents in biodistribution studies is an important consideration that can significantly impact the interpretation of results. One of the main advantages is that it allows for a clearer distinction between absorbed and unabsorbed radiotracer, thereby providing a more accurate assessment of systemic biodistribution. By eliminating the potential contribution from stomach and intestinal contents, researchers can obtain more reliable organ-specific activity and weight measurements. However, removing the contents may underestimate the total gastrointestinal retention of the radiotracer, particularly if a significant portion of the dose remain within the contents. This could lead to an inaccurate representation of the tracer bioavailability and absorption kinetics. Given these pros and cons, it is essential to carefully consider the study objectives when deciding whether to remove the contents. For instance, if the goal is to evaluate systemic distribution, removal may be beneficial. Conversely, if the focus is on understanding gastrointestinal transit or absorption, retaining the contents or counting them separately might be more appropriate.

### Perfusion

In select cases, for example when the radiotracer has a particularly long circulation half-life (nanoparticles, antibodies, albumin-binding radiotracers) or clear measure of activity accumulated in tissues versus remaining in blood in the organ is needed, transcardial perfusion before dissection can be conducted. The procedure should be carried out under terminal anesthesia according to the methods approved in the animal protocol. Typically, perfusion with buffered saline alone without fixative is sufficient for gamma counting purposes, but fixative can be added if downstream histological or immunohistochemical analyses are required. Blood sampling directly from the heart is usually feasible with a fine needle prior to dissection to install the perfusion cannula. For routine perfusions, investment in a good peristaltic pump operating smoothly at sufficiently low flow rates is advisable, as it speeds up the process and decreases interindividual variation in success and degree of the perfusion.

### Biodistribution Studies with Alpha and Pure Beta Emitters

When working with radionuclides that do not emit detectable gamma rays, especially alpha or pure beta emitters, careful selection of counting method and timing is essential. To solve this limitation, multiple approaches can be considered: i) indirect measurement through the detection of emissions in the decay chain of the desired radionuclide, or ii) more adapted and sensitive methods (e.g., liquid scintillation counting and gamma spectrometry).

Indirect measurements are applied for alpha-particle emitters, which decay through complex decay chain with multiple radioactive daughters (*t*_1/2_ µs to days) that emit various particles, including gamma rays. When the parent radionuclide, whose biodistribution is desired to be analyzed, does not emit gamma rays, a detectable daughter is commonly used to estimate the parent activity, calculated using the Bateman equation [18]:

$${A}_{d}\left(t\right)= {A}_{m}\left(0\right)\frac{{\lambda }_{d}}{{\lambda }_{d}-{\lambda }_{m}}\left({e}^{-{\lambda }_{m}t}- {e}^{-{\lambda }_{d}t}\right)*BR$$

where *A*_*m*_*(t)* and *A*_*d*_*(t)* are the activities of the mother and daughter at a time *t*, *λ*_*m*_ and *λ*_*d*_, their decay constants, and BR the branching ratio for the decay of the mother radionuclide into daughter. This equation defines their relationship under specific equilibrium conditions [19]: i) secular or ii) transient equilibrium.

**Fig. 4:**
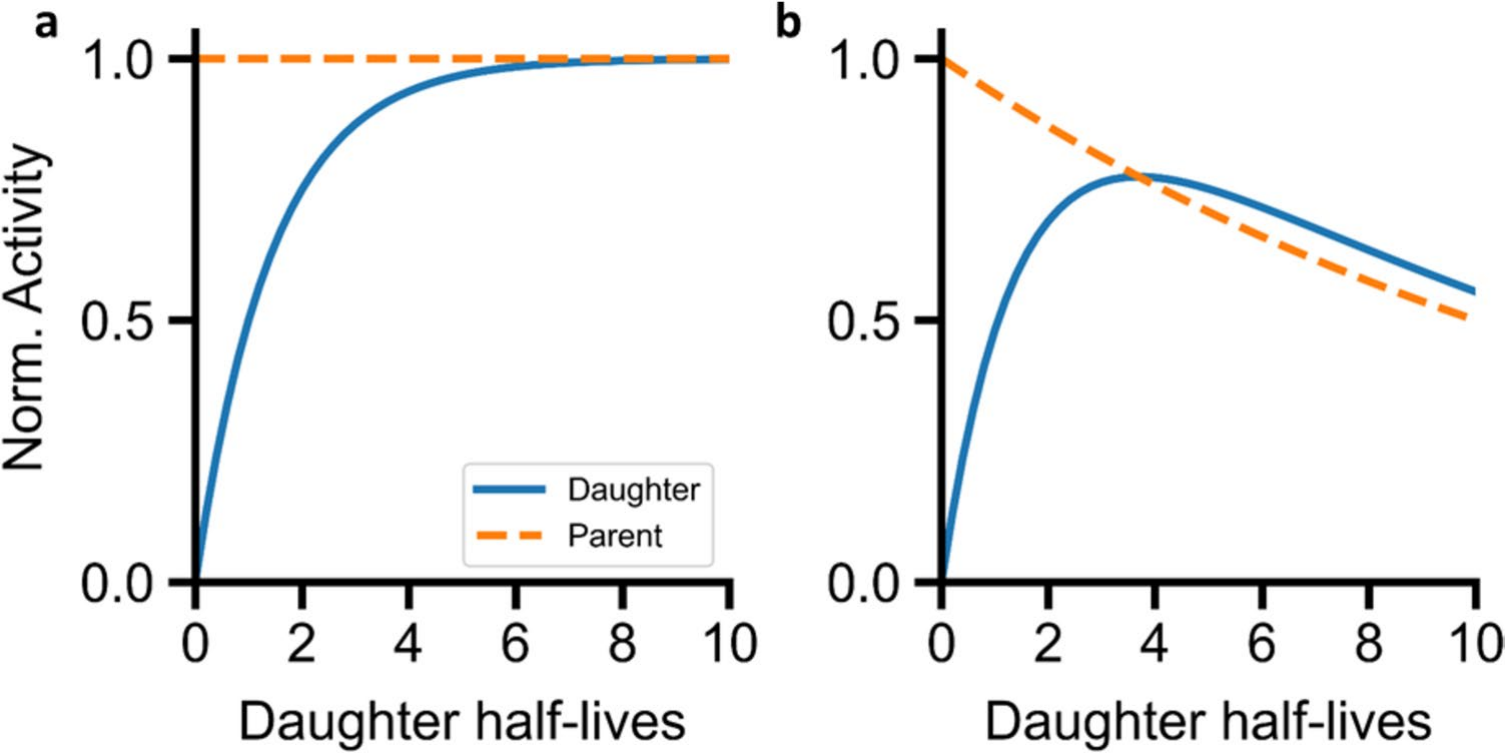
Schematic representation of (**a**) secular equilibrium considering λ_m_ ≈ 0 and a branching ratio of 1. After about 5 daughter half-lives, the activity of the daughter is equal to the activity of the mother and (**b**) transient equilibrium considering λ_m_ = 10 λ_d_ and a branching ratio of 1. Transient equilibrium is reached when the mother and the daughter decay following parallel curves

First, secular equilibrium (Fig. 4a): when the half-life of the mother radionuclide is so long that its activity remains nearly constant during observation, allowing λ_m_ ≈ 0. This simplifies the previous equation to:

$${A}_{d}\left(t\right)= {A}_{m}\left(0\right)\left(1- {e}^{-{\lambda }_{d}t}\right)*BR$$

After, one daughter half-life, ${A}_{d}= {~}^{1}\!\left/ \!{~}_{2}\right.{A}_{m}\left(0\right)*BR$

After, about five daughter half-lives, ${A}_{d}={A}_{m}\left(0\right)*BR$

Second, transient equilibrium (Fig. 4b): when the half-life of the mother is longer than the daughter’s, but λ_m_ can no longer be 0. The Bateman equation remains unsimplified. The daughter’s activity increases to reach a maximum value, and then decays at the same rate as the mother, defining transient equilibrium.

Consequently, samples from biodistribution studies that measure a daughter radionuclide’s activity indirectly should be timely corrected to ensure accurate correlation with the parent activity. The two following examples illustrate this situation.

Actinium-225 (alpha emitter, *t*_1/2_ = 9.92 days) decays to stable bismuth-209 through six short-lived daughters, emitting four alpha particles and two beta particles. Detectable gamma rays accompany the decays of francium-221 (218 keV, 11.4%) and bismuth-213 (440 keV, 25.9%). Most preclinical biodistribution studies measured ^225^Ac activity at secular equilibrium (24 h post-experiment) – between mother and daughters – using a gamma counter protocol based on ^221^Fr and ^213^Bi gamma emission [20–22]. A protocol using the sole ^221^Fr gamma energy window described by Castillo Seoane et al. demonstrated accurate ^225^Ac measurement with a reduced sample measurement waiting time of about 30 min [23]. Such protocols benefit radiopharmacy by enabling early activity reading during radiopharmaceutical purification using standard gamma counters instead of low-throughput gamma spectrometers. However, correction for ^213^Bi gamma ray down scatter in the ^221^Fr energy window is required. Interests in monitoring ^225^Ac daughter redistribution, especially ^213^Bi, led to a multiple time-point measurement protocol using the ^213^Bi gamma window with repeated measurements before reaching secular equilibrium between ^225^Ac and ^213^Bi (~ 5 h) [23].

For “in-house” generator-produced radionuclides like lead-212 (^212^Pb, *t*_1/2_ = 10.6 h), mother radionuclide breakthrough and its impact on biodistribution should be investigated. In ^224^Ra/^212^Pb generators, this is particularly challenging since the gamma peaks of ^212^Pb (239 keV) and ^224^Ra (241 keV) are distinguishable on a gamma counter. To determine ^224^Ra activity, samples are remeasured after ~ 5 days, allowing ^212^Pb activity from the initial elution to decay while breakthrough ^224^Ra reaches equilibrium with newly formed ^212^Pb [24].

For alpha emitters and radionuclides without detectable gamma emissions, such as pure beta emitters like ^90^Y, liquid scintillation counting is used. Radioactive tissue samples are dissolved in a liquid scintillation cocktail consisting of: 1) a solvent, usually aromatic, that absorbs the energy emitted by the radionuclide and 2) organic scintillators (phosphors or fluors) that converts absorbed energy into visible light. This approach requires the dissolution of the collected organs in strong acids, organic solvents, or mixtures before adding them to the scintillation cocktail. Colored solutions from tissue extracts can cause signal quenching, necessitating clearing agents like hydrogen peroxide to render the solutions colorless. In radiopharmaceutical biodistribution studies using tritiated or carbon-14-labeled tracers, scintillation counting remains a key technique alongside whole-body autoradiography [25].

Whether using gamma or liquid scintillation counting, the implementation of weighed formulation standards as presented in this protocol, enables activity determination in tissue samples without radionuclide-specific calibration curves. For scintillation counting, extra care must be taken to ensure the chemical composition of the standard solutions matches tissue extracts to maintain counting efficiency.

### Subsequent Assays with Biodistribution Samples

The gamma counting usually exposes the collected tissue samples to prolonged periods at room temperature, resulting in inevitable decomposition and spoiling of the samples. Therefore, care must be taken that samples remain suitable for subsequent immunohistochemical staining or other analyses. This might become a problem if the activity levels in the samples exceed the linear counting range of the instrument used, as might be the case with biodistribution studies from animals that have undergone imaging where typically higher levels of activity are needed. The samples should be stored in the fridge or freezer for the time they are not being counted, but usually subsequent assays are limited to destructive analyses (e.g., Western blotting). If counted within 1–2 h from collection, the samples can typically be snap-frozen or fixed as they emerge from the counter and used for autoradiography of tissue sections or immunohistochemistry. The feasibility of this is, however, strongly dependent of the type of analysis and biological target under study.

### Statistical Analysis

In biodistribution studies of radiopharmaceutical tracers, rigorous statistical analysis is crucial for interpreting distribution patterns and determining the significance of observed differences. Descriptive statistics, including mean, median, standard deviation, and coefficient of variation, provide a comprehensive overview of tracer uptake across various organs. Comparative analyses can be conducted using t-tests or analysis of variance (ANOVA) to evaluate differences between groups, while non-parametric tests, such as the Mann–Whitney U test, are appropriate for data that do not meet normality assumptions of the t-tests. Multivariate approach like multivariate analysis of variance (MANOVA) analysis facilitates the exploration of complex distribution patterns across multiple organs in different groups while considering the interrelationships between the variables. Correlation and regression analysis are also employed to investigate relationships between tracer uptake and physiological parameters. Additionally, time-activity curve analyses, such as area under the curve (AUC) and compartmental modeling, offer valuable information into kinetic parameters. In general, ensuring normality, homogeneity of variance, and appropriate corrections for multiple comparisons is essential for maintaining statistical validity. Analytical tools, such as GraphPad Prism, SPSS, and R, are highly recommended for implementing these statistical analyses effectively.

## Conclusions

Herein, we present a simple, straight-forward protocol for the determination of radiotracer distribution in excised tissue samples based on weighed standards of the injected formulation included in the activity measurement. This protocol constitutes a first step towards harmonization of the practices how biodistribution studies are carried out in molecular imaging and radiotherapy laboratories across the world, hopefully improving the reliability of the results obtained in the studies as well as prompting easier comparison of datasets. The accompanying biodistribution calculator tools are designed for facile input of the data and easy export of the data for any graph plotting software. Finally, since the protocol is based on injected tracer weight rather than generation of cumbersome calibration curves for detection instrumentation (which also drift), it can be with relative ease extended to studies using gamma spectrometry, liquid scintillation counting, or even non-radioactive detection methods, such as microwave plasma atomic emission spectroscopy (MP-AES) or inductively coupled plasma mass spectrometry (ICP-MS).

## Supplementary Information

Below is the link to the electronic supplementary material.

Supplementary file1 (PDF 284 KB)

Supplementary file2 (XLSX 81 KB)

Supplementary file3 (XLSX 112 KB)

## Data Availability

The spreadsheets for biodistribution calculation are freely available as the electronic Supporting Information for this article and the SimplyBiod© tool is available at https://simplybiod.streamlit.app/, with source code on GitHub repository (github.com/cecebert/SimplyBiod).
