# Supplementary material for: A Guide to Ex Vivo Biodistribution Studies with Radiotracers in Rodent Models
Source: Mol Imaging Biol. 2025 Oct 28;27(6):883–93. doi: 10.1007/s11307-025-02055-8 (PMC12804305; doi:10.1007/s11307-025-02055-8)
Supplement: Supplementary file 1 — Supplementary file1 (PDF 284 KB) [file 11307_2025_2055_MOESM1_ESM.pdf]

**SUPPLEMENTARY INFORMATION**

# A Guide to *Ex Vivo* Biodistribution Studies with Radiotracers in Rodent Models

Surachet Imlimthan[1], Cesare Berton[2], Sophie Poty[3], Jason P. Holland[2,*], Mirkka Sarparanta[1,*]

[1]Department of Chemistry, University of Helsinki, 00014 Helsinki, Finland

[2]University of Zürich, Department of Chemistry, 8057 Zürich, Switzerland

[3]Institut de Chimie Moléculaire de l'Université de Bourgogne (ICMUB), UMR CNRS 6302, Université de Bourgogne, 21000 Dijon, France

---

**Table of Contents**

Table S1 – An example of syringe preparation form

Table S2 – An example of animal injection sheet

Table S3 – An example of a general tissue list for *ex vivo* biodistribution studies

Table S4 – Acquired information and input flow of the biodistribution calculator

%ID $g^{-1}$ calculation

Standardized Uptake Value (SUV) calculation

**Table S1.** An example of syringe preparation form.

| **Date:** 23/10/2024 | | | | **Radiosynthesis batch no.:** 20241023-DT1 | | | |
|---|---|---|---|---|---|---|---|
| **Researcher:** Surachet Imlimthan | | | | **Tracer/Compound:** [$^{68}$Ga]Ga-DOTATATE | | | |
| **Activity concentration:** 20 MBq mL$^{-1}$ | | | | **Formulation/dose:** 100 μL in 0.9% isotonic saline | | | |
| **Syringe ID** | **Activity (Before injection, MBq or μCi)** | **Time** | **Syringe weight (Before injection, g or mg)** | **Residual activity (After injection, MBq or μCi)** | **Time** | **Syringe weight (After injection, g or mg)** | **Remark*** |
| 1 | 1.715 | 9:31 | 1.9804 | 0.018 | 11:48 | 1.8806 | |
| 2 | 1.721 | 9:32 | 1.9738 | 0.021 | 11:49 | 1.9229 | |
| 3 | 1.718 | 9:33 | 1.9849 | 0.020 | 11:50 | 1.8816 | |
| 4 | 1.744 | 9:34 | 1.9876 | 0.010 | 11:51 | 1.8759 | Standard |

**Corresponding animal ID and details can be added to the remark.*

**Table S2.** An example of animal injection sheet.

<table>
<tr><td colspan="4">Date: 23/10/2024</td><td colspan="4">Animal license no.: XXXXXX</td></tr>
<tr><td colspan="4">Researcher: Surachet Imlimthan</td><td colspan="4">Tracer/Compound: [$^{68}$Ga]Ga-DOTATATE (Batch no.: 20241023-DT1)</td></tr>
<tr><td colspan="4">Formulation: in 0.9% isotonic saline, 5% EtOH</td><td colspan="4">Injected volume: approx. 100 µL per dose</td></tr>
<tr><th>Syringe ID</th><th>Animal ID</th><th>Tail/ear marking</th><th>Time point</th><th>Injection time</th><th>End point</th><th>Body weight (g)</th><th>Tumor measurement (W and L in mm)*</th></tr>
<tr><td>1</td><td>M1</td><td>●</td><td>15 min</td><td>9:15</td><td>9:30</td><td>20.1280</td><td>W=3.07, L=3.08</td></tr>
<tr><td>2</td><td>M2</td><td>●</td><td>30 min</td><td>9:30</td><td>10:00</td><td>19.8572</td><td>W=3.94, L=4.11</td></tr>
<tr><td>3</td><td>M3</td><td>●</td><td>60 min</td><td>10:15</td><td>11:15</td><td>20.0017</td><td>W=3.70, L=4.50</td></tr>
</table>

**W for width (the smallest dimension) and L for length (the largest dimension) measured by Vernier calipers. Tumor volume =* $0.5\times L\times W^2$

**Table S3.** An example of a general tissue list for *ex vivo* biodistribution studies.

| Entry | Organ |
|---|---|
| Before opening the abdominal cavity | |
| 1 | Blood (cardiac puncture) |
| 2 | Tumor |
| After opening the thoracic and abdominal cavity (collecting from top to bottom) | |
| 3 | Heart |
| 4 | Lung |
| 5 | Gallbladder |
| 6 | Liver |
| 7 | Spleen |
| 8 | Pancreas |
| 9 | Stomach |
| 10 | Small intestines |
| 11 | Large intestines |
| 12 | Adrenal gland |
| 13 | Kidneys |
| 14 | Fat |
| 15 | Other internal organs of interest |
| 16 | Urine (from the bladder or $CO_2$ chamber) |
| 17 | Muscle |
| 18 | Tibia |
| 19 | Tail (injection site) |
| Head-and-neck region | |
| 20 | Skin (ear flap) |
| 21 | Occipital bone |
| 22 | Brain |

**Table S4.** Acquired information and input flow of the Microsoft Excel-based biodistribution calculator.

| Entry | Category | Item | Input location | Remark |
|---|---|---|---|---|
| 1 | General information | Name of radiotracer | C2 | |
| 2 | | Start of the counting time | C3 | Format: DD.MM.YYYY HH:MM<br>Note: All input values of activity are decay corrected to this time point. |
| 3 | | Dose calibrator background (MBq) | C4 | |
| 4 | | Radionuclide | C5 | Selection from a drop-down menu. Data provided are from the National Nuclear Decay Center, https://www.nndc.bnl.gov/nudat3/ |
| 5 | | Number of animals (n) | C8 | The Excel sheet contains sufficient space for a group of n = 15 animals. If additional space is required new rows & columns can be inserted |
| 6 | Animal, syringe, and injection details | Animal ID | B13 – B27 | |
| 7 | | Whole-body weight (g) | C13 – C27 | |
| 8 | | Syringe ID | D13 – D27 | |
| 9 | | Full syringe weight (g) | E13 – E27 | Before animal injection |
| 10 | | Empty syringe weight (g) | F13 – F27 | After animal injection |
| 11 | | Full syringe radioactivity (MBq) | H13 – H27 | Measured activity in the syringe before injection. Note the activity should be input without correction for background activity which is performed separately. Alternatively, if a manual correction for background is performed, the cell C4 should be zero. |

| | | | | |
|---|---|---|---|---|
| 12 | | Full syringe date and time | I13 – I27 | Recorded time of the dose calibrator assay of the full syringe. Format: DD.MM.YYYY HH:MM |
| 13 | | Animal injection date and time | J13 – J27 | Format: DD.MM.YYYY HH:MM |
| 14 | | Empty/residual syringe radioactivity (MBq) | K13 – K27 | Measured residual activity in the syringe after injection. See entry 11 for the note about background correction. |
| 15 | | Empty/residual syringe date and time | From L13 | Recorded time of the dose calibrator assay of the empty syringe.<br>Format: DD.MM.YYYY HH:MM |
| 16 | Standard syringe details | Full standard syringe weight (g) | E28 | Full syringe mass recorded before injection into a falcon tube containing water. |
| 17 | | Empty standard syringe weight (g) | F28 | Empty / residual syringe mass recorded after injection into a falcon tube containing water. |
| 18 | | Full standard syringe radioactivity (MBq) | H28 | Measured activity in the syringe before injection. See entry 11 for the note about background correction. |
| 19 | | Full standard syringe date and time | I28 | Format: DD.MM.YYYY HH:MM |
| 20 | | Injection time (into dilution tube) | J28 | Format: DD.MM.YYYY HH:MM |
| 21 | | Empty/residual standard syringe radioactivity (MBq) | K28 | Measured residual activity in the syringe after injection into the falcon tube containing water. See entry 11 for the note about background correction. |
| 22 | | Empty/residual standard syringe date and time | L28 | Format: DD.MM.YYYY HH:MM |
| 23 | Standard syringe | Empty 15-mL conical centrifuge tube weight (g) | C32 | |

| | | | | |
|---|---|---|---|---|
| 24 | dilution details | Tube + 9.9 mL of water weight (g) | C33 | Note: the precise volume of water used for the dilution is not important. The critical factor is to know the dilution mass obtained and from experiences, approximately 10 mL of water is sufficient to avoid saturating the gamma counter with the prepared standard count tubes. |
| 25 | | Tube + water + standard weight (g) | C34 | After injection of the standard syringe into the tube containing water. |
| 26 | Background counts | Blank counts (CPM) | From C41 to C44 | Note: Use non-decay corrected CPM (n = 4) from the gamma counter. Average background counts are calculated in cell C45 |
| 27 | Standard syringe calibration | Empty gamma counting tube weight (g) | From C51 to C54 | Use CPM data from the gamma counter that is decay-corrected to the start of gamma count measurement. Standards are counted in quadruplicate (samples A – D). Standards are used to calculate a value of CPM g$^{-1}$ of injectate, and also a calibration factor of CPM/MBq. In the event that no radiotracer sticks to the syringe, the CPM g$^{-1}$ values can be used but a full correction, accounting for residual activity in the individual syringes is given by using the CPM MBq$^{-1}$ correction factor. |
| 28 | | Full gamma counting tube weight (g) | From D51 to D54 | |
| 29 | | Normalized standard counts (CPM) | From F51 to F54 | |
| | | Counting standards | Rows 71 – 74 | Columns B – K |
| 30 | Gamma counting results of tissues | Counting blanks | Rows 75 - 78 | Data are recorded but inserted into a separate part of the Excel spreadsheet. See Entries 26–29 (above) |
| 31 | | Animal ID | Rows B79 – B318 | Animal assignment. All tissues collected for one individual animal are counted sequentially. The same order of tissues is used for each animal. |

| | | | | |
|---|---|---|---|---|
| 32 | | Sample assignment | Rows C79 – C318 | Reference: Table S3. This should be amended based on the user's preference for the counting order of tissues. |
| 33 | | Empty organ tube weight (g) | Rows D79 – D318 | Tube + cap weight, empty. |
| 34 | | Organ-filled tube weight (g) | Rows E79 – E318 | Tube + cap + tissue sample weight, full. The sample mass for each tissue is calculated in cells F79 – F318. |
| 35 | | Normalized sample counts (CPM) | Rows G79 – G318 | Note: Use CPM values decay-corrected to the start of the gamma count measurement.<br><br>Rows H79 – H318 calculated the background corrected CPM $g^{-1}$ values after subtracting the average background activity in CPM in cell C45, then dividing by the sample mass (cells F79 – F318).<br><br>Sample activity in units of MBq $g^{-1}$ are calculated in cells I79 – I318.<br><br>Tissue uptake in %ID $g^{-1}$ is calculated for each individual sample in cells J79 – J318.<br><br>SUV (by weight, in g) is calculated in cells K79 – K318. |

Note that the Excel spreadsheet provided four sections of output with the associated bar charts shown underneath each table.

**Blue table** (starting in the top left cell P10) gives the biodistribution data in units of %ID $g^{-1}$.

**Green table** (starting in the top left cell P48) gives the biodistribution data in units of SUV (by weight in grams).

**Red table** (starting in the top left cell AI10) gives the tumor-to-tissue contrast ratios calculated as dependent data sets within each individual animal, based on the biodistribution data in units of %ID $g^{-1}$.

**Purple table** (starting in the top left cell AI48) gives the tumor-to-tissue contrast ratios calculated as dependent data sets within each individual animal, based on the SUV data.

**Calculation of tissue uptake in units of %ID $g^{-1}$ and SUV**

**Calculated injected activity at injection time (MBq)**

= [(full syringe activity – dose calibrator BG) × Exp((–λ)(injection time – syringe preparation time) × 24h)] – [(residual syringe activity – dose calibrator BG) × Exp((–λ)(injection time – residual syringe time) × 24h)] (Eq.1)

Where: λ = decay constant and Δ time = day

**Calculated injected activity (MBq)**

= [Calculated injected activity at injection time (MBq, or Eq.1)] × Exp[(–λ)(start of counting time – injection time) × 24h] (Eq.2)

**Background (BG) corrected normalized counts per sample mass (CPM $g^{-1}$)**

= [Normalized sample counts (CPM) – Average non-decay corrected BG counts (CPM)]/[Sample mass (g)] (Eq.3)

**Standard (Std) calibration factor (CPM $MBq^{-1}$)**

= [Total normalized BG corrected counts per sample mass (CPM $g^{-1}$)]/[Calculated injected activity (MBq)/Calculated injected mass (g)] (Eq.4)

**Calibrated sample activity/mass (MBq $g^{-1}$)** = (Eq.3)/(Eq.4)

= [BG corrected normalized counts per sample mass (CPM $g^{-1}$)]/[Std calibration factor (CPM $MBq^{-1}$)] (Eq.5)

**%ID $g^{-1}$** = [(Eq.5)×100]/(Eq.2)

= [Calibrated sample activity per sample mass (MBq $g^{-1}$)×100]/[Calculated injected activity (MBq)] (Eq.6)

**SUV** = [%ID $g^{-1}$ (or Eq.6)]/[whole-body weight (g)/100] (Eq.7)
